# Population dynamics of multiple triplet excitons revealed from time-dependent fluorescence quenching of single conjugated polymer chains

**DOI:** 10.1038/s41598-018-37477-8

**Published:** 2019-01-28

**Authors:** Benjamin D. Datko, John K. Grey

**Affiliations:** Department of Chemistry and Chemical Biology, University of New Mexico, Albuquerque, NM 87131 USA

## Abstract

The advent of multiple exciton harvesting schemes and prolonging exciton lifetimes to improve performance attributes of solar cells based on conjugated organic materials presents some interesting challenges that must be overcome in order to realize the full potential of these strategies. This is especially important for applications involving multi-chromophoric conjugated polymers where interactions between multiple spin-forbidden triplet excitons can be significant and are mediated by chain conformation. We use single molecule spectroscopic techniques to investigate interactions between multiple triplet excitons and emissive singlets by monitoring time-dependent fluorescence quenching on time scales commensurate with the triplet lifetime. Structurally related conjugated polymers differing by heteroatom substitution were targeted and we use a stochastic photodynamic model to numerically simulate the evolution of multi-exciton populations following photoexcitation. Single chains of poly(3-hexylthiophene) (P3HT) exhibit longer-lived triplet dynamics and larger steady-state triplet occupancies compared to those of poly(3-hexylselenophene) (P3HS), which has a larger reported triplet yield. Triplet populations evolve and relax much faster in P3HS which only becomes evident when considering all kinetic factors governing exciton population dynamics. Overall, we uncover new guidelines for effectively managing multi-exciton populations and interactions in conjugated polymers and improving their light harvesting efficiency.

## Introduction

Conjugated organic polymers have demonstrated promise in solar cell applications but extreme heterogeneity and efficient intrinsic loss mechanisms^1,2^, such as rapid non-radiative excitation energy dissipation, are responsible for large disparities between measured and predicted efficiencies. There is now widespread interest for mitigating performance losses by generating multiple excitons per photon absorbed or extending exciton lifetimes^3–5^. Singlet fission–the generation of two triplet excitons from one singlet exciton–and heavy atom substitution to increase triplet character, respectively, have attracted the most attention^4,6–11^, but applications involving conjugated polymers are limited^12,13^. In fact, mechanistic studies of singlet fission have concentrated on *crystalline solids and small molecule arrays* with well-defined chromophore orientations. Furthermore, efforts to increase triplet exciton character and tune triplet interactions in polymers may be complicated by contributions from large vibrational displacements along high frequency modes that modulate spin-orbit coupling strength^14^.

Perhaps the most significant obstacle for effectively utilizing multi-exciton generation and harvesting strategies can be traced to the multi-chromophoric nature of polymers (i.e., many conjugated segments of varying length) and variable inter-chromophore coupling due to conformational heterogeneity^15^. For example, the longer lifetimes of triplets creates complex photophysical scenarios due to the presence of multiple excitonic states of different spin on many chromophore segments that interact over a broad range of time scales (e.g., ~10^−12^–10^−3^ s). Interestingly, previous pulse radiolysis studies found that isolated conjugated polymer chains can support a large number of triplets (~30) simultaneously^16^. While much of the current focus has emphasized elucidating triplet formation mechanisms on ultrafast time scales, relatively little is known about how populations of multiple triplet excitons evolve on longer time scales (i.e., comparable to triplet lifetimes).

Unfortunately, resolving multi-excitonic interactions at the materials level in polymers is complicated from a myriad of competing decay channels arising from intermolecular interactions and aggregation^12,17^. However, by dispersing polymers into inert glassy hosts, intermolecular interactions and packing heterogeneity are negated in addition to spatially confining excitonic kinetics and interactions to a single polymer chain (SPC). Single molecule spectroscopy can then be used to interrogate triplet population dynamics and interactions with emissive singlets by monitoring fluorescence quenching on nanosecond to millisecond time scales^18–21^. However, much of the earlier investigations of excitonic processes at the SPC level focused on energy transfer within the singlet manifold where funneling between chromophore sites typically dominates responses^22–26^. This regime is most prevalent when yields of spin-forbidden triplet excitons are small (<10%) although the presence of even one triplet can have significant consequences at the SPC level^27–29^. Triplet interactions with emissive singlets are commonly inferred from intermittency behavior of SPC fluorescence intensity transients in the form of flickering (i.e., fast cycling between “on” and “off” intensity levels) on millisecond time scales^30^ or blinking behavior due to sensitization of reactive oxygen species occurring on time scales of seconds^31^. More specialized single molecule spectroscopic tools have proven effective for exposing triplet interactions on faster time scales, such as, fluorescence correlation spectroscopy^32^ and excitation intensity modulation spectroscopy^33^. While these approaches can access population dynamics of excitonic configurations, kinetic models describing discrete excitonic interactions are much more complicated when multiple triplets are involved. For this reason, it is generally assumed that rates describing multi-excitonic triplet configurations and processes (i.e., triplet-triplet annihilation) reach a steady state condition immediately following photoexcitation^29^. In other words, triplet diffusion or the triplet-triplet annihilation rate constant are assumed to be infinitely fast leading to only one triplet at a time. This limit effectively reduces singlet-triplet interactions to a simple two-state description where the system spends most time in either the lowest energy triplet (T_1_) or ground electronic state (S_0_)^29^ although crucial details of multi-exciton interactions are lost.

We use single molecule excitation intensity modulation spectroscopy to probe triplet induced fluorescence quenching dynamics in SPCs of single poly(3-hexylthiophene) (P3HT) and poly(3-hexylselenophene) (P3HS), which have reported triplet yields exceeding 30%^34,35^. Although it is assumed that triplet formation follows a conventional perturbative mechanism, these systems are excellent models for understanding the full implications of heavy atom substitution at the SPC level in addition to resolving interactions between multiple triplets and triplet population dynamics. Quenching behavior is modeled by calculating the time-dependent probabilities of *n* = 0, 1, 2, … *nth* triplet population dynamics using a stochastic photodynamic model based on the Smith-Ewart differential difference equation originally developed to describe polymerization/emulsion kinetics^36–40^. We use the basic formalism of Barzykin and Tachiya^41^ and employ the approach of Birtwistle and co-workers^39^ to discretize and solve the Smith-Ewart model using the Gauss-Seidel iterative approach. Unlike effective two-state models mentioned earlier, this model incorporates finite triplet diffusion and triplet-triplet annihilation rate constants resulting in nonzero probabilities of multiple triplets at the SPC level. Simulations of triplet population and fluorescence quenching dynamics of P3HS and P3HT SPCs show good agreement with experiment when triplet-triplet annihilation rate constants are comparable to the natural first order triplet decay rate constant. Interestingly, despite larger reported triplet formation and decay rate constants in P3HS^34^, we observed larger steady-state probabilities of multiple triplets in P3HT chains. This unexpected result highlights the importance of accounting for all kinetic factors regulating population dynamics of multiple triplets that are difficult to obtain from ensemble level studies. Furthermore, singlet-triplet interactions were found to be much stronger in P3HS probably from substantial red-shifts in singlet exciton electronic transitions due to the heavier selenium heteroatom^42^ leading to improved spectral overlap between singlet donors and triplet acceptors. Overall, we demonstrate a robust and informative method for resolving the evolution of multiple triplet excitons to help bridge the gap in understanding of population dynamics on longer time scales.

## Results and Discussion

**Figure 1:**
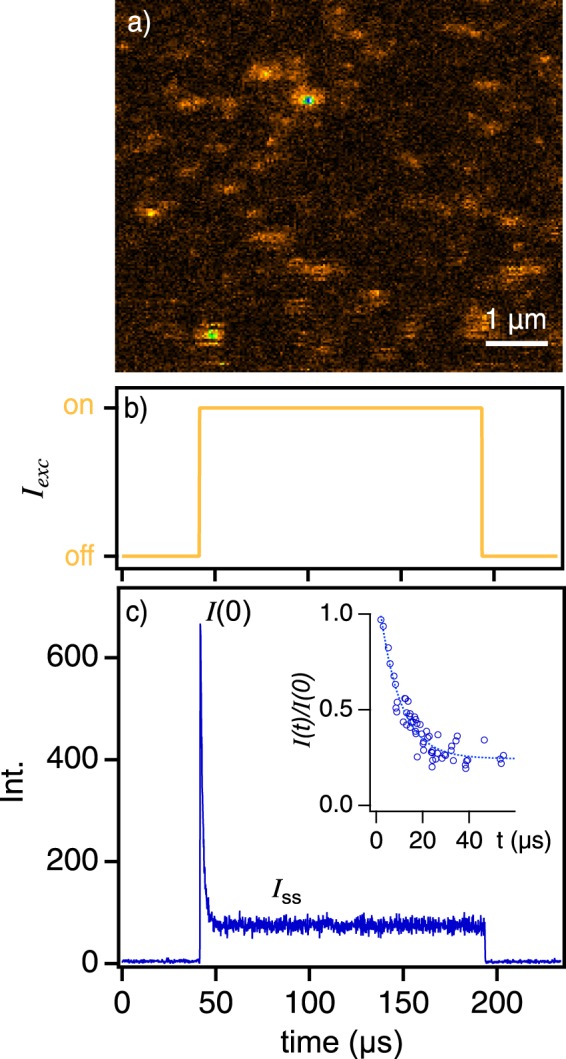
(**a**) Representative fluorescence image of SPCs dispersed in polystyrene matrices. (**b**) Rectangular laser excitation pulse waveform used to excite fluorescence in single polymer chains (top). (**c**) Example of fluorescence quenching dynamics in a single polymer chain with multiple triplets. Immediately after the laser turns on, intensities begin at an initial value, *I*(0), then, as triplet occupancies increase, decay to a non-zero steady state value, *I*_*ss*_, usually within or faster than the triplet lifetime. Inset: Histogram of quenching depths and decay times from over 40 P3HS molecules with an exponential decay fit as a guide for the eye.

Single molecule excitation intensity modulation spectroscopy was used to interrogate the presence of multiple interacting triplet excitons. Figure 1a shows a representative fluorescence image of well-dispersed P3HS SPCs in polystyrene. Importantly, intrinsically low fluorescence quantum yields (~10^−3^) due to efficient triplet formation lead to low signal-to-noise ratios and individual spots often show ‘streaking’ due to singlet quenching by triplets. SPC fluorescence emission and quenching dynamics are resolved by exciting individual molecules using sequences of rectangular shaped laser pulses displayed in Fig. 1b where pulse characteristics are tailored according to the expected triplet lifetimes^33,43^. Like other fluorescence-based single molecule probes, this technique relies on a loss of signal as an indicator of singlet-triplet interactions which, in the case of the target materials, can be very large leading to rapid quenching and low steady-state intensities. This effect can be seen in Fig. 1c which shows representative triplet-induced fluorescence quenching behavior of a single P3HS molecule in addition to average decay times and modulation depths from over 40 SPCs (inset). When the laser first turns on (t_0_) no triplets are present but, over time, triplet populations increase causing fluorescence quenching via singlet-triplet energy transfer (annihilation). Average triplet populations are also affected by intrinsic decay (i.e., reverse intersystem crossing) and triplet-triplet annihilation that depend on SPC structural and electronic factors^27,44^. Taken together, these processes cause the initial intensity, *I*(0), to decay to a steady-state level, *I*_*ss*_, and quenching depths can serve as a useful metric of both time-dependent triplet occupancies and the strength of singlet-triplet interactions. For ease of comparison with simulated quenching curves (vide infra), we report fluorescence quenching depths as the fraction of quenched fluorescence normalized to *I*(0), i.e., *I*(*t*)/*I*(0).

**Figure 2:**
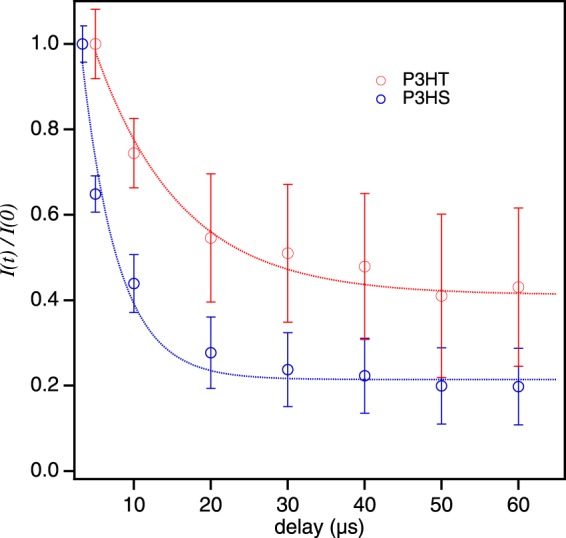
Experimental fluorescence quenching behavior of P3HS and P3HT comprised of ensemble averaged SPC data from a variable delay two-pulse approach recorded for various delay time intervals. Quenching dynamics are represented as *I*(*t*)/*I*(0) curves.

Because of the large variation in responses from SPCs of both polymers^43,45^, we used a modified version of excitation intensity modulation technique described earlier^45^ where two rectangular pulses of the same duration and intensity are temporally delayed and *I*(0) values of each are recorded with pulse delay time and converted into *I*(*t*)/*I*(0) curves. Figure 2 shows experimental single molecule fluorescence intensity quenching data obtained from P3HS (blue) and P3HT (red) single molecules of similar molecular weight prepared under the same conditions. The first pulse achieves a steady-state triplet population condition and the second pulse excites the SPC before triplet populations fully relax. Varying the time delay between the two pulses reveals triplet relaxation dynamics of SPCs that are comparable to quenching decay times directly from transients from low excitation intensities (e.g., Fig. 1). Responses in Fig. 2 are averaged over many (>40) SPCs of each polymer providing a better comparison to ensemble level measurements in addition to further exposing the role of the heteroatom on triplet formation efficiencies and singlet-triplet and triplet-triplet interactions. Average quenching depths of P3HS and P3HT SPCs range from ~60% to ~80% of *I*(0), respectively, (e.g., corresponding to *I*_ss_ values of ~0.4 and ~0.2, respectively). The larger average quenching depths and faster quenching dynamics of P3HS SPCs are consistent with larger triplet occupancies and singlet-triplet quenching rates but, it is not possible to ascertain the relative magnitudes of associated rate constants by inspection alone. The former depend on triplet formation yields as well as the triplet and fluorescence lifetimes, however, it is not straightforward to directly infer relative contributions of triplet-triplet annihilation from *I*(*t*)/*I*(0) curves^41,46^.

Barzykin and Tachiya^41^ had previously overcame the limitations imposed by assumptions of infinitely fast triplet-triplet annihilation by introducing an expanded stochastic photodynamic model to simulate the time-dependent evolution of multiple triplet populations from fluorescence intensity modulation data. Importantly, these authors only considered quenching behaviors from conjugated polymers with relatively low triplet yields (<10%) and, consequently, smaller populations of multiple triplet exciton configurations. We now simulate population dynamics of multiple triplets and their interactions with emissive singlets by adopting a similar approach as Barzykin and Tachiya, which benefits from the fact that triplet decay kinetics are orders of magnitude slower than singlet decay time scales^41^. The model accounts for the existence of multiple chromophores where each can occupy either S_0_, S_1_ or T_1_ states at any given time where only the numbers of each state determine the overall configuration and not the specific location on the SPC^41^. The kinetic scheme for triplet formation and decay is given as follows for n triplets on an SPC that follows the same format as the original Smith-Ewart model,

1 $$SP{C}_{n}\mathop{\longrightarrow }\limits^{{k}_{f,n}}SP{C}_{n+1}$$

2 $$SP{C}_{n}\mathop{\longrightarrow }\limits^{n\,{k}_{b}}SP{C}_{n-1}$$

3 $$SP{C}_{n}\mathop{\longrightarrow }\limits^{1/2n(n-1){k}_{TT}}SP{C}_{n-2}$$

where *k*_*f*,*n*_ is the forward rate constant for generating a triplet on an SPC with n triplets. It is assumed that triplet formation and decay mechanisms are governed by intersystem crossing via spin-orbit singlet-triplet mixing although the specific triplet generation mechanism is inconsequential. Now, the full form of *k*_*f*,*n*_ is,

4 $${k}_{f,n}=\frac{{k}_{exc}{k}_{ISC}{\tau }_{fl}}{1+{k}_{ISC}{\tau }_{fl}+\,n{k}_{QST}{\tau }_{fl}}$$

where *k*_*exc*_ is the excitation rate constant, *k*_*ISC*_ is the intersystem crossing rate constant, *τ*_*fl*_ is the fluorescence lifetime, and *k*_*QST*_ is the singlet-triplet quenching rate constant. Triplets decay to the S_0_ state via first order reverse intersystem crossing which is given by *k*_*b*_ (=${k^{\prime} }_{ISC}$), or, from triplet-triplet annihilation described by *k*_*TT*_, the pseudo first order rate constant in the Smith-Ewart description. We use reported values for *k*_*ISC*_, *τ*_*fl*_ and ${k^{\prime} }_{ISC}$ measured in dilute solutions or in solid dispersions which are held invariant to extract *k*_*TT*_ and *k*_*QST*_ estimates from fluorescence quenching curves. P3HS has much larger reported *k*_*ISC*_ values of 3 × 10^10^ s^−1^ compared to P3HT of ~1 × 10^9^ s^−1^ due to larger singlet-triplet spin-orbit mixing^34,47,48^. Likewise, fluorescence lifetimes in P3HS are ~26 ps that were estimated from singlet exciton lifetimes from stimulated emission decays as well as from lifetimes measured at the SPC level^34,43^. Reported *τ*_*fl*_ values in solvated P3HT chains are ~500 ps but care must be taken to ensure no appreciable aggregation exists resulting in a larger contribution from a fast decay component associated with torsional relaxation within aggregate π-stacks^49^. Triplet lifetime $(1/{k^{\prime} }_{ISC})$ estimates were generated from previous single molecule intensity modulation investigations yielding values of ~2 × 10^5^ s^−1^ and ~1 × 10^4^ s^−1^, for P3HS and P3HT, respectively^43,45^.

The time-dependent probability of n triplets, *P*_*n*_(t), can now be obtained by solving the Smith-Ewart differential difference equation^37,38,40^,

5 $$\begin{array}{c}\frac{d}{dt}{P}_{n}(t)={k}_{f,n-1}{P}_{n-1}(t)-[{k}_{f,n}+n{k}_{b}+1/2n(n-1){k}_{TT}]{P}_{n}(t)+\,{k}_{b}(n+1){P}_{n+1}(t)\\ \,\,\,\,+[1/2(n+1)(n+2){k}_{TT}]{P}_{n+2}(t)\end{array}$$

**Figure 3:**
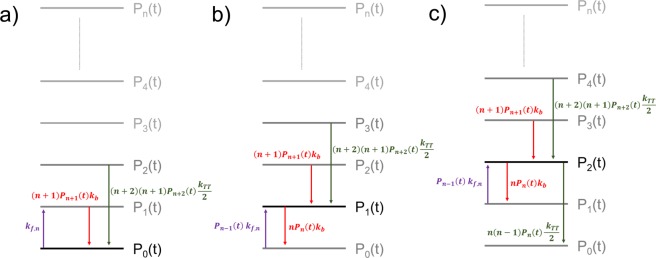
Schematic representation of the model showing the influence of the three principle rates on the probability of zero, one, and two triplets in the conjugated polymer, panels (a–c) respectively.

And the time-dependent fluorescence intensity, *I*(t), is expressed as^41^,

6 $$I(t)=I(0)\sum _{n=0}^{\infty }\,\frac{{P}_{n}(t)}{1+\,n\,{k}_{QST}{\tau }_{fl}}$$

where *I*(0) is the initial (normalized) intensity (t = 0). Details for solving Eq. 5 are provided in the Supplemental Information section and Fig. 3 shows a diagram describing the probabilities and transition rates involving zero (a), one (b), and two (c) triplets on an SPC. Three principle rates drive the formation of any state, namely, (i) gain of one triplet from intersystem crossing, (ii) loss of one triplet from reverse intersystem crossing, and (iii) loss of two triplets through annihilation. For any number of triplets, each probability is governed by the same principle rates. At t = 0 (i.e., when the laser turns on), the probability of having zero triplets decreases due to a constant forward (zero order) rate constant, *k*_*f*,0_. As triplet occupancies increase, new rates of loss processes become important. In addition to population losses through reverse intersystem crossing, which varies linearly with the current number of triplets, triplet-triplet annihilation becomes operative for more than one triplet proportional to the square of the current occupancy. Importantly, this model implies that, if probabilities of any two states are equal and non-zero, the current state (*P*_*n*_(*t*)) will always gain infinitely more triplets from annihilation of a higher state (i.e., *P*_*n*+2_(*t*)) than losing triplets from annihilation to a lower state, (i.e., *P*_*n*−2_(*t*)).

It is first instructive to consider a useful approximation where *k*_*f*,*n*_ is constant (*k*_*f*,*n*_ ~ *k*_*f*,0_ = *k*_*exc*_ *k*_*ISC*_ *τ*_*fl*_). This regime enables estimates of *P*_*n*_(*t*) at long times, or, *P*_*n*_(∞) without the need to solve the Smith-Ewart equation exactly^41^, which is helpful for estimating steady-state multi-triplet populations using only known photophysical constants (vide supra). In an earlier study of P3HS SPCs, we used this assumption and found non-zero probabilities of more than one triplet on an SPC when *k*_*TT*_ was similar to ${k^{\prime} }_{ISC}$^43^. These preliminary estimates of steady-state triplet populations revealed that the assumption of infinitely large *k*_*TT*_ is not valid in SPCs with large triplet yields (large *k*_*ISC*_). This result can be checked easily by using the condition *k*_*TT*_ ≫ ${k^{\prime} }_{ISC}$. For example, rapid triplet-triplet annihilation to just one triplet on an SPC requires unrealistically large values of *k*_*QST*_ to achieve *I*(*t*)/*I*(0) comparable to experiment^41^. In order to accurately reproduce experimental fluorescence quenching behavior, the Smith-Ewart equation must be solved numerically to calculate *P*_*n*_(t). We applied the general method of Birtwistle *et al*.^39^ to solve the Smith-Ewart model and estimate *k*_*TT*_ and *k*_*QST*_ with significant nonzero multi-triplet populations.

**Figure 4:**
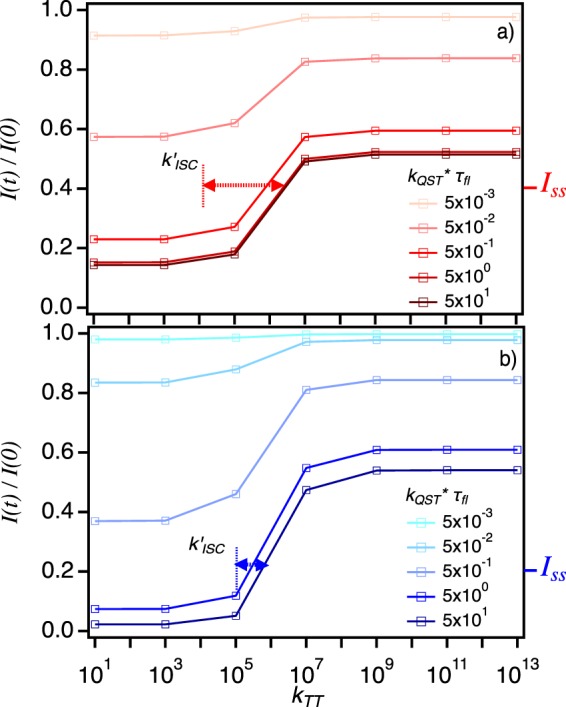
(**a**) Fluorescence intensity quenching of P3HT (**a**) and P3HS (**b**) calculated from assuming a constant product of the singlet-triplet quenching rate constant (*k*_*QST*_) while varying the triplet-triplet annihilation rate constant (*k*_*TT*_). We assume a lower limit of the latter by referencing to the reverse intersystem crossing rate constant (${k^{\prime} }_{ISC}$). Arrows represent the likely range of *k*_*TT*_ values at the average steady state intensity (*I*_*ss*_).

Unfortunately, the difficulty in extracting reliable values of *k*_*TT*_ in either P3HS or P3HT at the ensemble level usually due to the disappearance of triplet signatures or ambiguous dynamics^47^ necessitates first exploring realistic ranges of possible values. This was accomplished by varying *k*_*TT*_ and comparing calculated quenching behavior to experiment for fixed values of *k*_*QST*_. Figure 4 shows *I*(t)/*I*(0) behaviors for varying *k*_*TT*_ at fixed *k*_*QST*_ values referenced to the reported fluorescence lifetimes, *τ*_*fl*_. Estimates of *k*_*QST*_ from other polymers at the SPC level were typically in the range of ~10^8^–10^10^ s^−1^ which provides an additional benchmark for establishing realistic limits of this parameter^29^. We imposed a lower limit on *k*_*TT*_ corresponding to ${k^{\prime} }_{ISC}$ for each polymer and horizontal arrows in Fig. 4 depict the expected range of *k*_*TT*_ based on averaged experimental steady-state intensities (*I*_*ss*_) values of ensemble quenching curves in Fig. 2. Comparison of quenching behaviors reveals a larger sensitivity to *k*_*TT*_ in P3HS based on experimental quenching depths indicating stronger singlet-triplet interactions. It is also interesting to note that triplet lifetimes decrease drastically with increasing triplet densities due to faster triplet-triplet annihilation^16^. Although the presence of multiple triplets in P3HT and P3HS is apparent, it is doubtful that the relatively low excitation intensities used here (ca. 10–100 W/cm^2^) ever enter regimes where the bimolecular annihilation process vastly exceeds linear triplet decay mechanisms.

The results also reveal that an increase in *k*_*QST*_ accompanies increases in *k*_*TT*_ values due to the fact that fewer triplets are present. Furthermore, we show in the following that experimentally measured quenching depths and dynamics place fundamental limits on *k*_*QST*_ that support our assumption of *k*_*TT*_ is comparable to ${k^{\prime} }_{ISC}$. It is also interesting to note that when *k*_*QST*_ is small in both polymers (*k*_*QST*_ * *τ*_*fl*_ < 10^−2^), *I*(*t*)/*I*(0) behaviors are practically invariant of *k*_*TT*_ indicating no interactions between singlets and triplets regardless of their occupancies. This regime could represent the case when SPC conformations are extended and low excitation intensities (i.e., small *k*_*exc*_), thus requiring excitons to diffuse over longer distances, and triplet lifetimes are short compared to *τ*_*fl*_. Additionally, if *τ*_*fl*_ is relatively small (e.g., P3HS), larger triplet occupancies or singlet-triplet interactions (viz. *k*_*QST*_) are needed to produce appreciable quenching.

**Figure 5:**
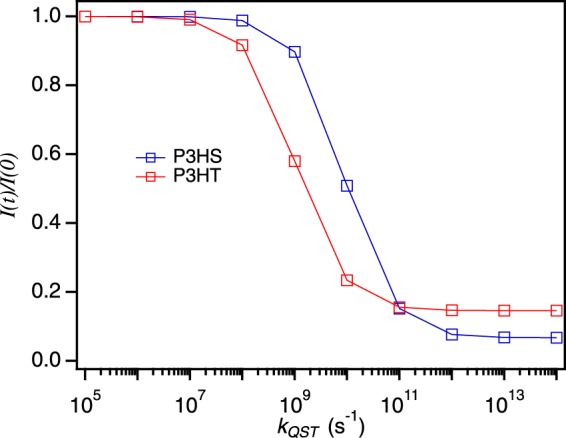
Fluorescence intensity quenching of P3HS and P3HT calculated from the assumption of ${k^{\prime} }_{ISC}$ = *k*_*TT*_ while varying *k*_*QST*_.

Using the assumption, ${k}_{TT}={k^{\prime} }_{ISC}$, *k*_*QST*_ is next varied over several decades to assess the sensitivity of singlet-triplet interactions and their effect on *I*(*t*)/*I*(0). Figure 5 shows simulated fluorescence quenching depths and, from inspection, larger *k*_*QST*_ are required for P3HS consistent with stronger singlet-triplet interactions. This effect can be rationalized by the fact that the heavier selenium atom causes significant red-shifting of singlet electronic transitions^42^ which should result in better spectral overlap between singlet donor emission and triplet acceptor absorption and, consequently, larger *k*_*QST*_. Furthermore, based on experimental *I*(*t*)/*I*(0) curves, P3HS displays substantially larger quenching depths which implies that *k*_*QST*_ must be sufficiently large compared to the excited state lifetime. Additional insights for validating the choice of *k*_*TT*_ and *k*_*QST*_ values can be obtained from triplet formation quantum yields (Φ_ISC_). According to experimental estimates of *k*_*ISC*_ and *τ*_*fl*_ from previous ensemble measurements provided earlier and in Table 1, Φ_ISC_ values for P3HT are ~0.5 compared to P3HS of ~0.8 leading to the expectation of larger triplet occupancies in the former. However, triplet decay by reverse intersystem crossing back to S_0_ and triplet-triplet annihilation is faster in P3HS, reflected in estimates of ${k^{\prime} }_{ISC}$ of 2 × 10^5^ s^−1^ compared to 1 × 10^4^ s^−1^ of P3HT^43^. Rapid triplet population decay from larger triplet-triplet annihilation and reverse intersystem crossing rate constants in P3HS drastically reduce triplet occupancies despite their larger formation yields, requiring larger values of *k*_*QST*_ to achieve fluorescence quenching depths comparable to experiment.

**Table 1:** Fluorescence quenching simulation parameters for P3HT and P3HS.

Parameter	P3HT	P3HS
*k*_*exc*_ (s^−1^)	1 × 10^6^	1 × 10^6^
*k*_*ISC*_ (s^−1^)	1 × 10^9^	3 × 10^10^
${k^{\prime} }_{ISC}$ (s^−1^)	1 × 10^4^	2 × 10^5^
*k*_*QST*_ (s^−1^)	5 × 10^8^	8 × 10^10^
*k*_*TT*_ (s^−1^)	1 × 10^4^	2 × 10^5^
*τ*_*fl*_ (s)	500 × 10^−12^	26 × 10^−12^

**Figure 6:**
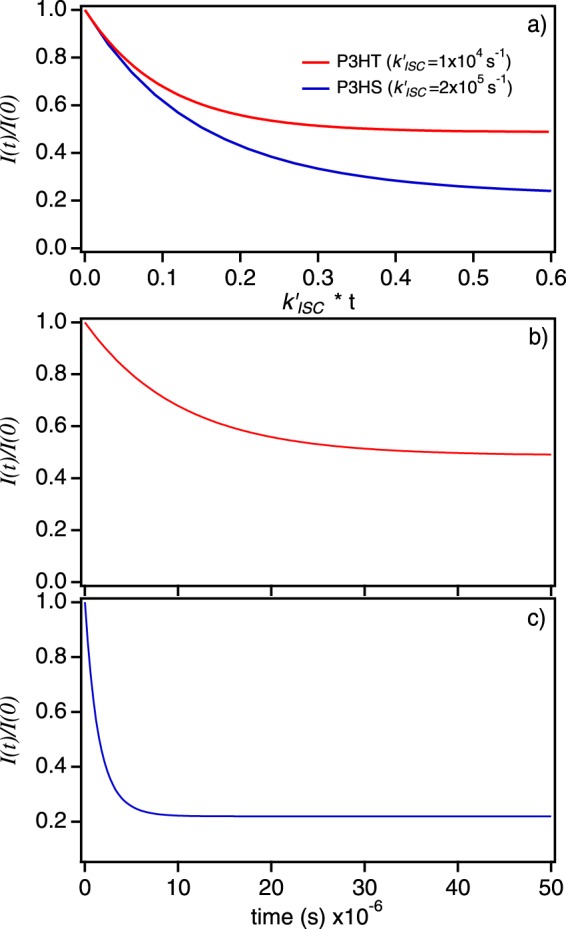
Fluorescence quenching of P3HS and P3HT SPCs. (**a**) I(t)/I(0) plots scaled to ${k^{\prime} }_{ISC}$ for each polymer. Time-dependent quenching depth curves for P3HT (**b**) and P3HS (**c**).

We now simulate time-dependent fluorescence quenching (*I*(*t*)/*I*(0)) behavior and triplet populations for P3HS and P3HT SPCs using Eqs 5 and 6 based on estimates of *k*_*TT*_ and *k*_*QST*_ from Figs 4 and 5. Table 1 summarizes all parameter values used in the simulations and results are displayed in Fig. 6 and compared to experimental data in Fig. 2. Values of *k*_*exc*_ were generated assuming an absorption cross-section of ~10^−15^ cm^2^ for each polymer with an excitation power density of ~10 W/cm^2^ at 568 nm. To facilitate comparison between polymers we plot *I*(t)/*I*(0) curves by multiplying the simulation time step by ${k^{\prime} }_{ISC}$ for each polymer in addition to displaying fluorescence quenching with time. Similar to experiment, P3HS shows faster quenching dynamics and larger quenching depths as expected for larger *k*_*QST*_ and multiple triplets. As predicted in Figs 4 and 5, shallow and slower quenching in P3HT can result from either inefficient singlet quenching by triplets (lower *k*_*QST*_) or faster triplet-triplet annihilation as well as shorter-lived singlet exciton (S_1_) states. Because measured *τ*_*fl*_ values are over an order of magnitude larger in P3HT compared to P3HS, the latter possibility can be easily ruled out. Earlier work on SPCs containing only light atoms reported *k*_*QST*_ values within the same range as generated here although these studies also assumed infinitely fast *k*_*TT*_. Since this latter regime allows only one triplet quencher at a time, larger *k*_*QST*_ are needed to achieve substantial quenching depths and faster quenching dynamics. For example, comparison of quenching behavior between earlier studies and ours indicate that exceptionally large *k*_*QST*_ values (e.g., >10^12^ s^−1^) would be necessary to produce observed quenching depths found in P3HS and P3HT chains. This observation confirms that triplet-triplet annihilation must be held finite in systems with larger triplet yields and occupancies.

**Figure 7:**
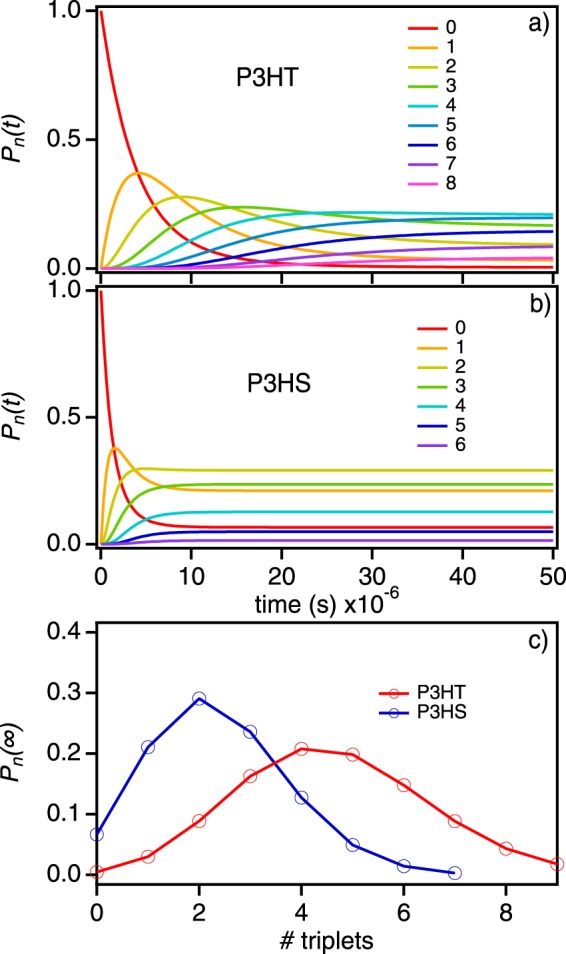
Time-dependent populations of triplets in P3HT (**a**) and P3HS (**b**) for multiple triplet configurations. (**c**) Steady-state triplet populations (*P*_*n*_(∞)) for each polymer.

Time-dependent triplet occupancies for *I*(*t*)/*I*(0) curves (Fig. 6) are next shown in Fig. 7 depicting the evolution of multiple triplet configurations over the excitation pulse duration (~100 μs). Comparing triplet population dynamics to quenching behaviors reveals some interesting trends that are not immediately obvious from reported photophysical constants. First, steady-state triplet populations are much larger in P3HT despite having lower Φ_ISC_ values. This effect arises from slower triplet-triplet annihilation and reverse intersystem crossing as well as the longer excited state lifetimes. The shallow quenching depths and slower dynamics also indicates that singlet-triplet interactions are significantly weaker in P3HT which, assuming a resonant energy transfer mechanism, is probably from lower spectral overlap compared to P3HS. Secondly, Fig. 7 reveals that P3HS triplet occupancies reach a steady-state condition much faster than P3HT. Calculating *P*_*n*_(t)–allowing for up to 15 triplets–reveals that P3HS triplet dynamics are complete by ~3 ± 1 µs compared to ~25 ± 6 µs in P3HT resulting in steady-state triplet populations (*P*_*n*_(∞)) of ~2 ± 2 triplets for P3HS compared to ~4 ± 3 triplets on P3HS chains. The lower values of *P*_*n*_(∞) from P3HS chains are a consequence of both faster triplet relaxation and triplet-triplet annihilation for multiple triplet configurations coupled with a very short effective excited state lifetime. In contrast, weaker singlet-triplet interactions, slower triplet-triplet annihilation and longer-lived excited states in P3HT result in larger triplet occupancies despite having lower triplet formation yields.

Plots of *P*_*n*_(t) dynamics in Fig. 7 reveal useful insights into the roles of triplet lifetimes and interactions affecting triplet population decay that are essential for properly interpreting *I*(*t*)/*I*(0) behavior of both systems. This was particularly apparent in the case of large quenching depths and faster quenching dynamics in P3HS despite having smaller triplet occupancies at longer times. This result appears counterintuitive when only considering triplet formation yields which have been shown earlier to depend on the heteroatom. It is also informative to point out how the choice of *k*_*TT*_ and *k*_*QST*_ used in the simulations of *I*(*t*)/*I*(0) curves and *P*_*n*_(t) dynamics in Figs 6 and 7, respectively, can be validated from comparison with experiment. For example, we considered scenarios where *k*_*TT*_ > ${k^{\prime} }_{ISC}$ and *k*_*TT*_ < ${k^{\prime} }_{ISC}$ and varied *k*_*QST*_ to tune quenching responses that can be compared directly to experiment. We set an upper limit of ~10^11^ s^−1^ which is comparable to the fastest observed singlet-triplet quenching rate constant reported in conjugated polymers^29^. When *k*_*TT*_ > ${k^{\prime} }_{ISC}$ triplet occupancies shift to lower values (i.e., *P*_*n*_(∞) shifts to lower values) requiring larger *k*_*QST*_ to achieve the same quenching depth seen experimentally (see Supplemental Information). However, upon comparison with experimental *I*(*t*)/*I*(0) curves, poor agreement arises from quenching dynamics behaviors, i.e., simulated quenching dynamics are much faster. On the other hand, by allowing *k*_*TT*_ < ${k^{\prime} }_{ISC}$, *k*_*QST*_ decreases drastically as shown in Fig. 5 due to much larger triplet occupancies (i.e., *P*_*n*_(∞) shifts to larger values). Although previous pulse radiolysis work on larger polymer chains found evidence that an SPC can support up to 30 triplets^44^, it is doubtful that such large steady-state occupancies are possible here since both polymers are relatively small (ca. 30 KDa).

Lastly, photophysics of polymers are highly dependent on the SPC conformational qualities and we expect that values of *k*_*TT*_ and *k*_*QST*_ to fluctuate with sample preparation conditions (viz. solvent) and from molecule-to-molecule. In fact, previous single molecule spectroscopic investigations of singlet-triplet interactions found drastic variations in fluorescence responses with molecular size^32^ and order^45^. We expect that SPC conformational qualities should have a large impact on *k*_*TT*_. Here, we do not expect either P3HS or P3HT to be able to self-aggregate which should attenuate triplet diffusion and triplet-triplet annihilation. Further examination of this effect is beyond the scope of the present paper due to large variations in solubility of both polymers although large variations in fluorescence quenching often appear with very subtle changes in chain conformation and order^45,50^. In addition to triplet-triplet annihilation, self-aggregation in larger polymer chains has been proposed to increase singlet-triplet interactions^32,51,52^. However, reliably sorting out the dominant energy transfer mechanism has proven difficult. Nonetheless, long-range, Forster-type energy transfer mechanisms should not be affected by conformation as much as exchange mediated, Dexter-type energy transfer.

## Conclusions

We have demonstrated new perspectives of kinetic factors governing the occupancies of triplets in conjugated polymers with large triplet yields on time scales relevant to optoelectronic devices. This feature, along with the multi-chromophoric nature of polymers, increases the likelihood of multiple excitonic species existing on an SPC at any given time. Our simulations have revealed several critical points that should be taken into consideration when designing excitonic materials for optoelectronic applications. Specifically, multi-exciton generation and heavy atom substitution approaches to generate and harvest many triplets. Perhaps the most noteworthy of these involves the kinetic competition between triplet formation and decay mechanisms that determine triplet occupancies on longer time scales. This effect was most pronounced in P3HS where, despite larger triplet yields, faster relaxation through first and second order processes lowered triplet occupancies compared to P3HT. We expect these processes in addition to singlet-triplet interactions to be strongly dependent on the SPC conformation, which can be simulated by adjusting the associated rate constants. Overall, our approach can now provide a clearer link between triplet formation (e.g., singlet fission or increased triplet admixture) on sub-picosecond time scales and triplet interactions on much longer time scales.

## Methods

The synthesis and characterization of P3HS and P3HT (~30 KDa, PDI~1.2) were described in detail previously^42^. Dilute solid dispersions of each polymer were prepared by dissolving in chlorobenzene (~10^−9^ M) which was added to a polystyrene solution. Thin films were deposited by spin coating on rigorously cleaned glass coverslips and sealed with an aluminum layer (~100 nm) from vacuum deposition. Single molecule excitation intensity modulation experiments were performed on a scanning confocal based microscope spectrometer described earlier^45^. Briefly, SPCs were excited by a series of rectangular laser pulses (568 nm, Intra-action) and fluorescence intensity trajectories were averaged many thousands of cycles with an multi-channel analyzer (Fast Com Tec). Triplet quenching dynamics were also measured by using a delayed two-pulse excitation arrangement where one rectangular pulse excites the SPC to produce a steady-state triplet population distribution followed by a second identical pulse. By delaying the second pulse in time, initial intensities (I(0)) can be used as a metric of triplet relaxation in the absence of light. Details of this approach were provided in ref.^45^. Time-dependent triplet populations and triplet-induced fluorescence quenching behaviors were simulated by solving the Smith-Ewart equation using the approach of Birtwistle and co-workers^38,39^. Details of the computational approach are provided in the Supplemental Information Section.

## Supplementary information

Supp. Info.

## Data Availability

Single molecule fluorescence intensity transients and triplet simulation scripts are available upon request.
