# Supplementary material for: Population dynamics of multiple triplet excitons revealed from time-dependent fluorescence quenching of single conjugated polymer chains
Source: Sci Rep. 2019 Jan 28;9:817. doi: 10.1038/s41598-018-37477-8 (PMC6349865; doi:10.1038/s41598-018-37477-8)
Supplement: Supplementary file 1 — Supp. Info. [file 41598_2018_37477_MOESM1_ESM.pdf]

Population dynamics of multiple triplet excitons revealed from time-dependent fluorescence quenching of single conjugated polymer chains

*Benjamin D. Datko and John K. Grey**

Department of Chemistry and Chemical Biology, University of New Mexico, Albuquerque, NM 87131.

*jkgrey@unm.edu

SUPPLEMENTAL INFORMATION

Numerical Solution to the Smith-Ewart Difference equation

We employ the approach as Birtwistle and coworkers[1, 2] to solve the Smith-Ewart equation in MATLAB. The full form is solved using the iterative approach of Gauss-Seidel, and discrete time intervals. The discrete form then becomes,

$$\begin{aligned}\frac{P_{n,q+1}-P_{n,q}}{h} &= (1-\theta)\Big[\big(P_{n-1,q}-P_{n,q}\big)k_{f,n}+\big\{(n+1)P_{n+1,q}-nP_{n,q}\big\}k_b \\ &+\big\{(n+2)(n+1)P_{n+2,q}-n(n-1)P_{n,q}\big\}\frac{k_{TT}}{2}\Big] \\ &+\theta\Big[\big(P_{n-1,q+1}-P_{n,q+1}\big)k_{f,n}+\big\{(n+1)P_{n+1,q+1}-nP_{n,q+1}\big\}k_b \\ &+\big\{(n+2)(n+1)P_{n+2,q+1}-n(n-1)P_{n,q+1}\big\}\frac{k_{TT}}{2}\Big]\end{aligned} \tag{1}$$

Changing from continuous to discrete time intervals was made through substitution of $t = qh$, where q is the incremental timestep and h is the time interval for the step. Here, $P_n(t)$ was replaced with $P_{n,q}$ and $\frac{d}{dt}P_n(t)$ was replaced with $P_{n,q+1}$. The value θ a weighting parameter between zero and one. The value is set fixed to 0.5 as the authors prescribed in their discussion of the stability of the iterative procedure.[1] Eq. 1 can be solved with the following initial conditions,

$$t=0\begin{cases} P_{0,0}=1 \\ P_{n>0,0}=0,\ \ n=1,2,\dots,n_{max} \\ P_{0,1}=\dfrac{\theta\{k_bP_{1,1}+k_{TT}P_{2,1}\}+\frac{1}{h}-(1-\theta)k_{f,n}}{\frac{1}{h}+\theta k_{f,n}}\end{cases} \tag{2}$$

It is important to note the terms $P_{n-1,q}$ and $P_{n-1,q+1}$ are set to zero when $n-1<0$. The full form of the Smith-Ewart equation in our model includes an infinite number of triplet states, and therefore

must be truncated at a given n_{max}, which an appropriate limit is assessed by evaluating the ranges of second order parameters while holding first order parameters fixed. The simulations usually converge quickly with a tolerance of 10^{-8} within first hundred iterations and typical parameters used for both P3HS and P3HT are $n_{max} = 200$, $q_{max} = 300$, $tol = 10^{-8}$, and $h = 1/(100 * k_b)$.

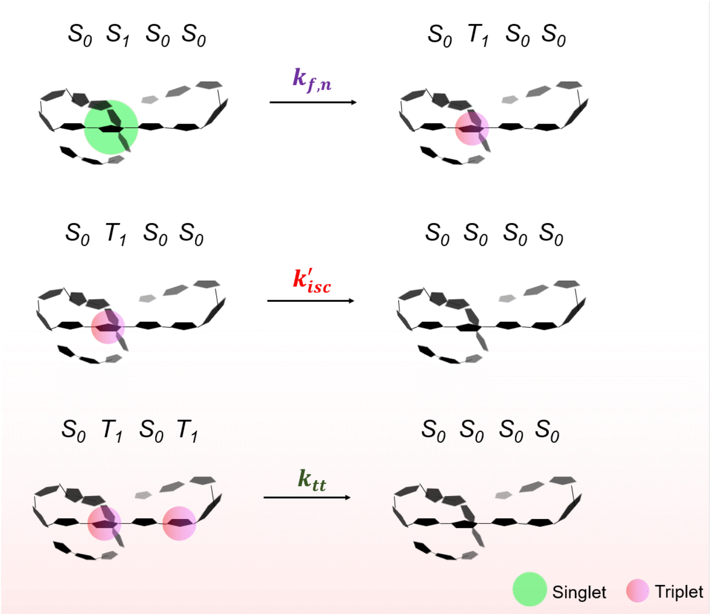

Figure S1. Cartoon schematic of triplet formation, first and second order decay processes.

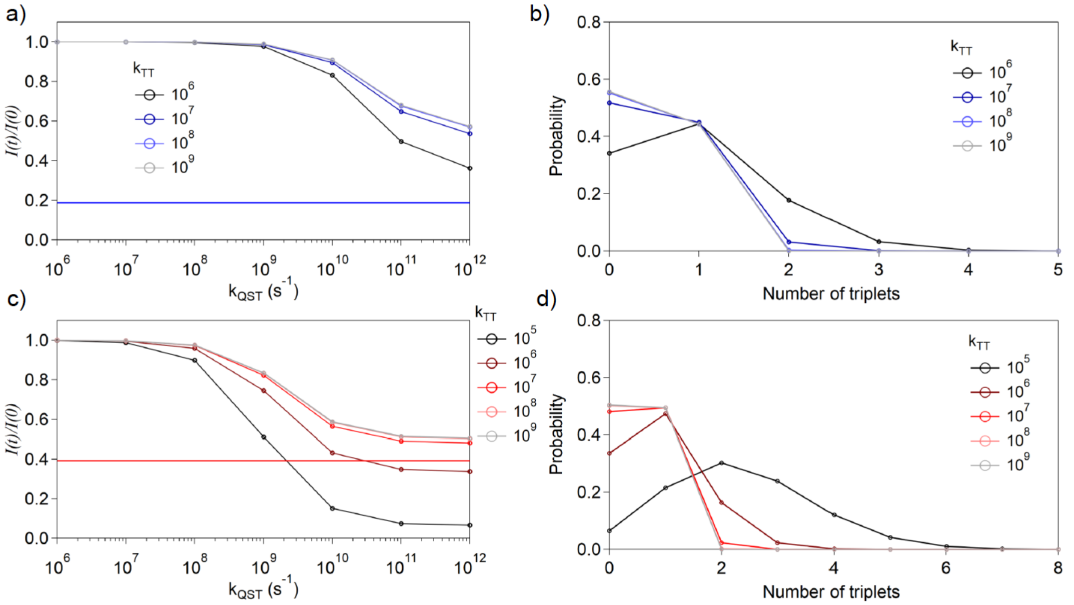

Figure S2. Effect of variable triplet-triplet annihilation constants (i.e., k_{TT}) on quenching depths and steady-state triplet populations and quenching dynamics in P3HS and P3HT chains.

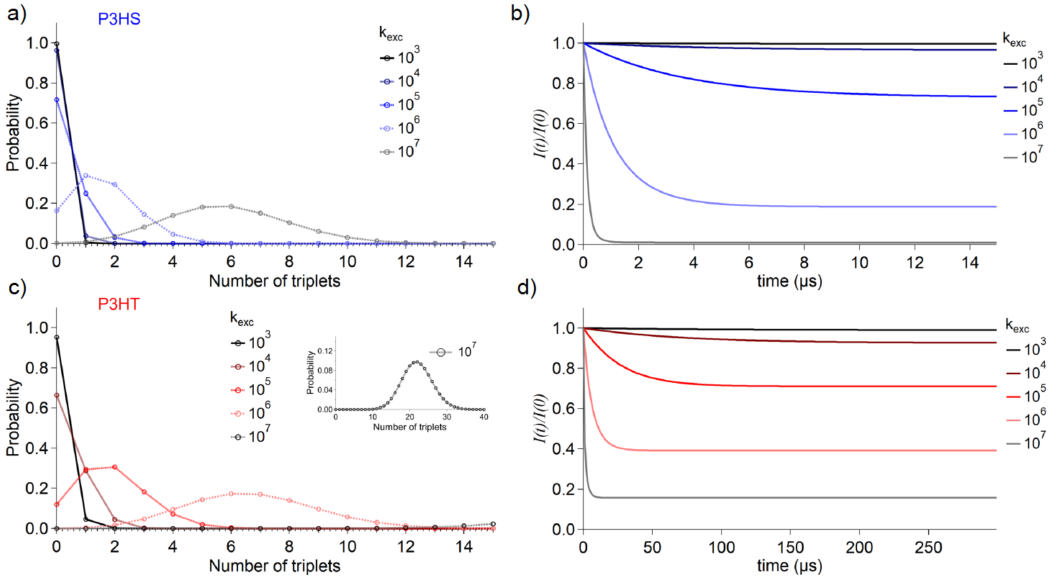

Figure S3. Effect of variable excitation intensities (i.e., k_{exc}) on triplet populations and quenching dynamics in P3HS and P3HT chains.

References

1. Ballard, M. J.; Gilbert, R. G.; Napper, D. H., Improved Methods for Solving the Smith-Ewart Equations in the Steady-State. *J Polym Sci Pol Lett* **1981**, *19*, 533-537.
2. Birtwistle David, T., Theory of Compartmentalised Free-Radical Polymerisation Reactions. Part 5. *J. Chem. Soc., Faraday Trans. 1* **1981**, *77*, 1351-1358.
